# The Opportunistic Pathogen *Staphylococcus warneri*: Virulence and Antibiotic Resistance, Clinical Features, Association with Orthopedic Implants and Other Medical Devices, and a Glance at Industrial Applications

**DOI:** 10.3390/antibiotics13100972

**Published:** 2024-10-15

**Authors:** Stefano Ravaioli, Andrea De Donno, Giulia Bottau, Davide Campoccia, Alessandra Maso, Paolo Dolzani, Paulraj Balaji, Francesco Pegreffi, Maria Daglia, Carla Renata Arciola

**Affiliations:** 1 Laboratorio di Patologia delle Infezioni Associate all’Impianto, IRCCS Istituto Ortopedico Rizzoli, Via di Barbiano 1/10, 40136 Bologna, Italy; andrea.dedonno@ior.it (A.D.D.); giulia.bottau@ior.it (G.B.); davide.campoccia@ior.it (D.C.); 2 Quality Control in GMP, IRCCS Istituto Ortopedico Rizzoli, 40136 Bologna, Italy; alessandra.maso@ior.it; 3 Laboratorio di Immunoreumatologia e Rigenerazione Tissutale, IRCCS Istituto Ortopedico Rizzoli, Via di Barbiano 1/10, 40136 Bologna, Italy; paolo.dolzani@ior.it; 4 PG and Research Centre in Biotechnology, MGR College, Hosur 635130, TN, India; balaji_paulraj@yahoo.com; 5 Department of Medicine and Surgery, School of Medicine and Surgery, “Kore” University of Enna, 94100 Enna, Italy; francesco.pegreffi@unikore.it; 6 Unit of Recovery and Functional Rehabilitation, P. Osp. Umberto I, 94100 Enna, Italy; 7 Department of Pharmacy, University of Napoli Federico II, Via D. Montesano 49, 80131 Naples, Italy; 8 International Research Center for Food Nutrition and Safety, Jiangsu University, Zhenjiang 212013, China; 9 Laboratory of Immunorheumatology and Tissue Regeneration, Laboratory on Pathology of Implant Infections, IRCCS Istituto Ortopedico Rizzoli, Via di Barbiano 1/10, 40136 Bologna, Italy; 10 Department of Medical and Surgical Sciences (DIMEC), University of Bologna, Via San Giacomo 14, 40126 Bologna, Italy

**Keywords:** *Staphylococcus warneri*, *Staphylococcus*, orthopedic infections, orthopedic implant infections, medical device-associated infections, opportunistic infections, bacteriocins, one health, antibiotic resistance, bacteria in industrial applications

## Abstract

In recent decades, the risk of developing opportunistic infections has increased in parallel with the ever-increasing number of people suffering from chronic immunosuppressive diseases or undergoing prosthetic surgery. *Staphylococcus warneri* is a Gram-positive and coagulase-negative bacterium. Usually found as a component of the healthy human and animal microbiota of the skin and mucosae, it can take on the role of an opportunistic pathogen capable of causing a variety of infections, ranging from mild to life-threatening, not only in immunocompromised patients but even, although rarely, in healthy people. Here, in addition to a concise discussion of the identification and distinguishing features of *S. warneri* compared to other staphylococcal species, a systematic overview of the findings from case reports and clinical studies is provided. The paper highlights the virulence and antibiotic resistance profiles of *S. warneri*, the different clinical contexts in which it has proven to be a serious pathogen, emphasizing its ability to colonize artificial prosthetic materials and its tropism for musculoskeletal and cardiovascular tissues. Some original data on orthopedic implant infections by *S. warneri* complement the discussion. Finally, from a different perspective, the paper addresses the possibilities of industrial exploitation of this bacterium.

## 1. Introduction

*Staphylococcus warneri* is a Gram-positive, coagulase-negative, catalase-positive, and oxidase-negative bacterium, closely related to *Staphylococcus epidermidis* and other Coagulase-Negative Staphylococci (CoNS) [1,2,3]. The name comes from Arthur Warner Jr., the patient from whom this bacterium was originally isolated by Wesley E. Kloos and Karl H. Schleifer in 1975 [4]. The main characteristics of *S. warneri* observed by Kloos and Schleifer in their collection of 38 isolates were the following: 0.5 to 1.2 µm in diameter, non-motile and non-spore-forming; with cells occurring mainly in pairs and singly, sporadically in tetrads; resistant to lysozyme, slightly resistant to lysostaphin; with weak or no hemolytic and variable bacteriolytic activity [4]. The colonies were small, entire, circular, smooth, sticky; 80% of the colonies were pigmented and bright yellow-orange or slightly yellowish at the edges, while 20% were non-pigmented, white-grey colonies [4].

**Figure 1:**
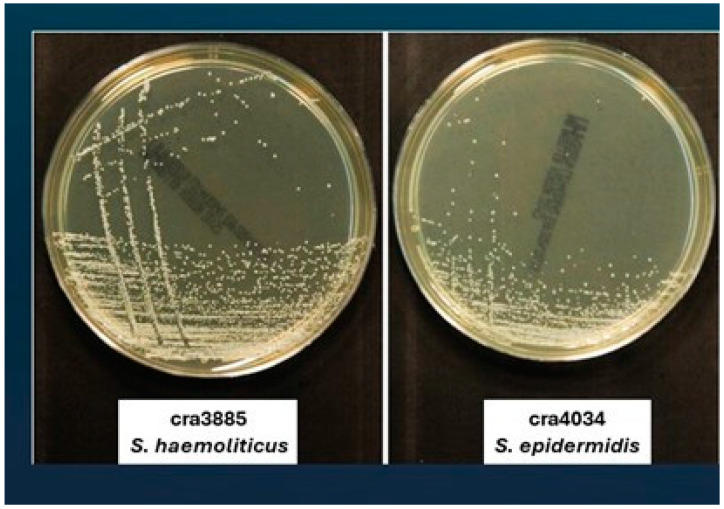

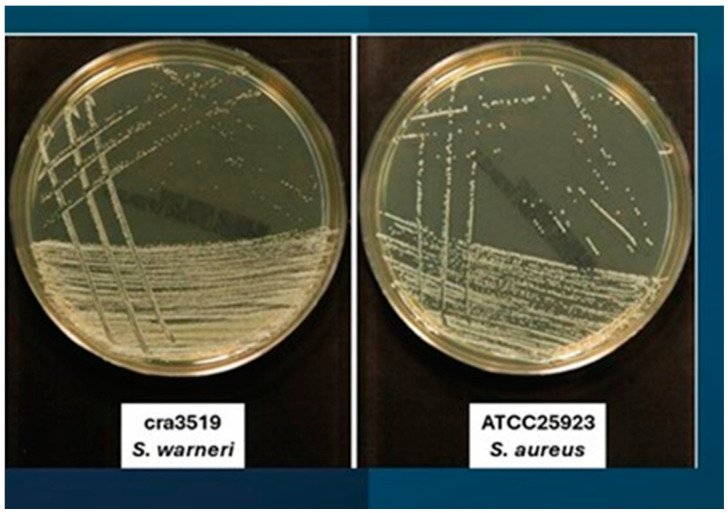
Size of 24-h colonies on Mueller Hinton agar from clinical isolates of different staphylococcal species. *S. haemolyticus*, *S. epidermidis*, *S. warneri,* and *S. aureus*. All bacterial isolates come from orthopedic implant infections and belong to the collection of the Research Laboratory on Pathology of Implant Infections of the Rizzoli Orthopedic Institute.

Figure 1 shows the typical small colonies from a clinical isolate of *S. warneri* in comparison with those of clinical isolates belonging to other staphylococcal species (photo taken in the Research Laboratory on Pathology of Implant Infections of the Rizzoli Orthopedic Institute).

*S. warneri* has been detected in approximately 50% of healthy adults [5], where it is a component of the normal skin microbiome as a common saprophyte. It is also present in the mucosae [6], such as those of the nasal [7] and oral cavities [8].

*S. warneri* is generally not a particularly frequent species [9], but it appears to be the second [10,11] and fourth most isolated species among staphylococcal microorganisms in infections associated with orthopedic implants (unpublished data from the Research Laboratory on Pathology of Implant Infections of the Rizzoli Orthopedic Institute) and in community and hospital settings, respectively [12]. In 2005, after counting clinical isolates belonging to staphylococcal species (included in the biobank of clinical bacterial isolates from orthopedic infections of the Research Laboratory on the Pathology of Implant Infections), *S. warneri* appeared in sixth position after *S. aureus* (the leading etiological agent of orthopedic implant infections), *S. epidermidis* (second), *S. hominis* (third), *S. haemolyticus* (fourth), and *S. capitis* (fifth), sorting the different species by prevalence [13]. 

*S. warneri* has been found in foods of animal origin, such as raw dairy products, chicken, pork, beef, and wild rabbit meat [6,14,15,16].

The main attitude of this important opportunistic pathogen is as a potential etiological agent of different types of infections [17]. Indeed, it has been reported in native valve endocarditis, vertebral osteomyelitis, discitis, urinary tract infections, neonatal sepsis, meningitis associated with ventriculoperitoneal shunt, subdural empyema, and bacteremia [1,4,7,18,19,20,21,22,23,24]. Over the past two decades, *S. warneri* has been considered a new emerging pathogen that leads to severe invasive infections in immunocompromised patients, even in the absence of foreign bodies [25], and in association with prostheses [26] or other medical devices, such as artificial heart valves and catheters, respectively [27,28].

The species *S. warneri* has also been reported in the veterinary field. Examples include abortion in cattle [29], and meningoencephalitis in dogs [30]. Furthermore, in rainbow trout, *S. warneri* has been found as a commensal with the potential to become an indirect pathobiont that helps pathogens, such as *Vibrio anguillarum*, to colonize and grow [31]. In addition, like (or perhaps more than) other species of staphylococci hitherto considered of lesser importance because they were classified as saprophytes or mild pathogens, *S. warneri* appears to be an increasingly resistant and multi-resistant species to antibiotics. It is worth noting, as one of the many examples we will consider that, in a collection of CoNS from cattle and buffalo with mastitis in Egypt, resistance to more than three antibiotics was found only in *S. haemolyticus* and *S. warneri* [14]. Considering that *S. warneri* causes severe infections associated with bacteremia and septicemia, along with the high rate of antibiotic resistance and other pathogenic characteristics, such as biofilm production and the ability to internalize into different types of human cells, we may argue the importance of further studying this bacterium. To date, knowledge of the molecular epidemiology of *S. warneri* and the pathogenetic mechanisms through which it can cause infection is still lacking or not exhaustive [25].

In another context, attention should be drawn to the fact that *S. warneri* is also used for industrial purposes to obtain special flavors in some fermented foods.

The aim of this paper is to present an overview of the techniques for its identification, the clinical aspects of *S. warneri*, its pathological characteristics, the issues of antibiotic resistance and therapy, and an excursus on how it is implicated in the industrial field.

## 2. Identification

*S. warneri* is commonly identified using biochemical galleries or commercial kits, such as the Api Staph identification system or ID32 Staph test. However, validation using molecular techniques is required, due to probable misidentifications with other staphylococcal species. *S. warneri* has a similar biochemical profile to *S. aureus,* the coagulase negative *S. epidermidis,* and other CoNS species, such as *S. haemolyticus* and *S. hominis*, making misidentification between *S. warneri* and the aforementioned staphylococcal species extremely likely [1,2,3,12,32,33]. Indeed, in our laboratory, some isolates which, through biochemical characterization, had been attributed to staphylococcal species other than *S. warneri* turned out to be *S. warneri* upon genotyping with RiboPrinter^®^. Vice versa, by genotyping with RiboPrinter^®^, we recognized the belonging to staphylococcal species other than *S. warneri* of isolates that had been identified as *S. warneri* through biochemical characterization (Table 1; data published for the first time). Kawamura et al. (1998) identified correctly only 7% of *S. warneri* by conventional biochemical tests and proposed DNA–DNA hybridization as a promising method suitable for the genetic identification of *S. warneri* [3]. *S. warneri* is also identified by 16S rRNA gene sequencing [34,35,36,37,38,39] or by in situ hybridization with oligonucleotide probes targeting 16S rRNA [40]. Iwase et al. (2007) proposed a simple species-specific real-time PCR assay based on the *sodA* gene for the identification and quantitative analysis of *S. capitis*, *S. haemolyticus* and *S. warneri* [41]. Other authors have described a simple, rapid and accurate PCR method for the identification of *S. warneri* based on nucleotide sequences of thermo-nuclease (*nuc*) genes [12,33,42].

No molecular epidemiology studies on *S. warneri* are reported in the literature, as the genetic or molecular techniques have been aimed exclusively at its identification. Molecular characterization of *S. warneri* clinical isolate collections could be useful in elucidating the molecular epidemiology, phylogenetics, and molecular evolution of this emerging pathogen. Campoccia et al. (2010) determined by automated ribo-printing, in a collection of 26 clinical isolates of *S. warneri*, five distinct ribo-groups, one of which was composed of fourteen isolates, and found that two ribo-groups were resistant to the four β-lactams tested, namely penicillin (PEN), ampicillin (AMP), cefazolin (CFZ), and cefamandole (FAM) [43].

## 3. Clinical Aspects

*S. warneri* can cause various diseases in adults, neonatal, and pediatric populations, as well as in the veterinary field. Moreover, *S. warneri* produces staphylococcal toxins that affect the food chain and are responsible for foodborne infections.

Below we present the most interesting clinical cases of *S. warneri* infection.

### 3.1. Bacteraemia

*S. warneri* bacteremia characterized by positive blood cultures, without other causes of fever or clinical decline, has been detected in multiple circumstances. In the present section, we recall the observations that emerged from two important studies published in the 1990s (the first referred to was published in 1997 and the second in 1992).

A retrospective study conducted at the Royal Children’s Hospital of Melbourne (Victoria, Australia) recognized 30 examples of *S. warneri* bacteremia in children, with *S. warneri* viability and verification according to the methods described by Kloos and Schleifer in 22 episodes, 12 of which reported as significant examples of bacteremia [20]. In that study, PFGE of chromosomal DNA demonstrated related band patterns in 20 of the 22 strains. The authors reported that, at their institution, *S. warneri* was the second most common CoNS species isolated from blood after *S. epidermidis*. Moreover, importantly they highlighted that intravascular devices (ten central venous catheters, two peripheral intravenous cannulas, and one peripheral intra-arteria-line) were present in all their patients with significant bacteremia.

Another previous study conducted at the Long Island Jewish Medical Center (New York City, USA) had reported 27 patients with blood cultures positive for *S. warneri*, fourteen then classified as significant examples of bacteremia (five in children and nine in adults) [18]. Among these cases, eight were associated with central venous catheters, five were of an unknown source, and one was associated with a native valve endocarditis [18].

### 3.2. Native Valve Endocarditis (NVE)

The epidemiology of infective endocarditis (IE) has changed over time due to factors like an aging population and the use of cardiac devices and valves [44].

Kamath et al. (1992) found a fulminant *S. warneri* native valve endocarditis in one patient with unknown valvular heart disease and with an underlying immunosuppressive condition [18]. Diaconu et al. (2019) reported a rare case of native valvular endocarditis caused by *S. warneri* in a 79-year-old man with valvular disease and without any previous risk factors [45]. The patient had heart failure due to mitral valve perforation but did not show any signs of infection. Diagnosis was confirmed by echocardiography and positive blood cultures [45].

El Nakadi and El Nakadi (2021) reported a 28-year-old woman with severe aortic regurgitation and thickening of the aortic cusp without typical vegetations [46]. After suffering from aphasia, despite antibiotic therapy being conducted and even with negative blood cultures for infective endocarditis, surgery became necessary. The aortic valve appeared normal, but the noncoronary and right coronary cusps were covered with thick, granular tissue, and *S. warneri* was isolated from the valve [46].

Dan et al. (1984) and Bhardwaj B. et al. (2016) highlighted two rare cases of endocarditis caused by *S. warneri*, which demonstrated its ability to cause endocarditis even in individuals without typical predisposing factors [47,48]. In the first study, a patient with unknown risk factors developed native valve endocarditis with *S. warneri* infection [47]. The patient had previously undergone hardware insertion in the ankles and had a scalp laceration, which could have facilitated the bacterial seeding on the mitral valve [47]. The second study involved a patient who developed endocarditis on a regular aortic valve after a vasectomy [48]. He experienced symptoms such as epididymitis, headaches, blurred vision, fatigue, anorexia, and chest pain. Blood cultures confirmed *S. warneri* infection, and an echocardiogram revealed left ventricular enlargement and severe aortic regurgitation. Surgical exploration showed destruction of the aortic valve and the presence of a small amount of vegetation [48].

Yamamoto et al. (2020) reported a 59-year-old patient with a 28-year history of type 1 diabetes mellitus and with a native valve endocarditis (NVE) caused by *S. warneri* [49]. Prior to this diagnosis, the patient had never experienced any health problems, although he had been diagnosed with chronic, severe asymptomatic aortic regurgitation (AR) sixteen years before. The patient had insignificant laboratory abnormalities and transient weight loss, but no other symptoms until the onset of acute heart failure. Upon admission, three sets of blood cultures were positive for *S. warneri*. Diagnostic tests revealed extensive vegetation on both the mitral and aortic valves, requiring the patient to undergo a double valve replacement [49].

Kurihara et al. (2021) described a 72-year-old Asian woman with a history of mitral valve regurgitation presenting with intermittent fever, general fatigue, and infective endocarditis (IE) caused by *S. warneri* on the native mitral valve [50]. Prior to admission, the patient had not received any medication for 10 years and the previous echocardiography did not show any vegetation on the valves. However, all four sets of blood cultures confirmed the presence of *S. warneri*, and a trans-esophageal echocardiography revealed a 5 mm mobile vegetation on the anterior cusp of the mitral valve, along with mitral valve regurgitation. The patient had conditions of spondylitis and cerebellar infarction. This case highlights the development of infective endocarditis in a patient with a history of mitral valve regurgitation, despite no previous evidence of vegetation on the valves [50].

In a recent case report, Alawad et al. (2022) presented a rare case of endocarditis caused by *S. warneri* in a 45-year-old male patient [51]. The patient presented symptoms of rigor, chest pain, and difficulty breathing, along with intermittent fever for the past three weeks. The patient had a medical history of well-controlled type 2 diabetes and coronary artery disease. Although initial blood cultures were negative, a chest X-ray revealed lung infiltrates. The patient was then admitted and treated for community-acquired pneumonia, but his fever persisted, and a new heart murmur developed. Further investigations using transthoracic echocardiography showed mitral regurgitation, and repeated blood cultures identified *S. warneri* as the causative agent. The patient’s condition worsened as trans-esophageal echocardiography revealed a perforated mitral valve leaflet with severe regurgitation. Subsequently, the patient underwent surgical repair of the mitral valve, and a methicillin-susceptible *S. warneri* isolate was cultured from the infected valve tissue [51].

### 3.3. Orthopedic Implant Infections

In the aforementioned collection of bacterial isolates from orthopedic implant infections of the Research Laboratory on the Pathology of Implant Infections (Rizzoli Orthopaedic Institute, Bologna), *S. warneri* turns out to be the third prevalent CoNS species, the fourth prevalent staphylococcal species, and the ninth most prevalent bacterial species.

We identified and characterized by automated ribotyping (Riboprinter^®^ Microbial Characterization System) 48 *S. warneri* clinical isolates, 75% of those coming from orthopedic implant-associated infections (Table 2; unpublished data). Formerly, Campoccia et al. (2010) displayed that 69% of the 26 *S. warneri* clinical isolates from orthopedic infections came, more precisely, from infections associated with implants [43]. Specifically, 44% were associated with hip implants, 28% with knee implants, 22% with internal fixation systems, and one infection involved an allogenic bone graft.

### 3.4. Vertebral Osteomyelitis

Karthigasu et al. (1986) reported an 81-year-old woman with lower thoracic back pain, which after radiological examinations showed a crush fracture of T9 secondary to osteoporosis, and subsequent scans revealed vertebral osteomyelitis [52]. The lesion was aspirated by needle biopsy, and microscopical examination of the material obtained showed a moderate number of leucocytes and few Gram-positive cocci. The specimen was cultured and a pure growth of a coagulase-negative Staphylococcus was then identified as an *S. warneri* isolate [52].

### 3.5. Septic Arthritis

Septic arthritis caused by indolent infections is challenging and difficult to diagnose promptly. In recent years, an increase in cases of septic arthritis caused by *S. warneri*, particularly in patients with prosthetic devices, has been reported. Legius et al. (2012) reported a 38-year-old male patient who developed indolent septic arthritis due to *S. warneri* [24]. The patient initially had redness, which resolved without medical treatment. The symptoms of septic arthritis appeared ten weeks after a traffic accident, which resulted in head-cerebral trauma and intracranial hypertension. The patient required temporary ventricular drainage. The swelling in the right knee was observed at the beginning of rehabilitation, but no evidence of infection was found in the synovial fluid. Further diagnosis was conducted using needle arthroscopy, which revealed a hyperemic and hypertrophic synovial membrane. Biopsies of the synovial tissue confirmed the presence of *S. warneri*. *S. warneri* was hypothesized to have invaded the bloodstream through the catheter during the patient’s stay in the intensive care unit [24].

### 3.6. Discitis

Discitis is a bacterial infection of the intervertebral discs that can occur from surgery or via the bloodstream. It is important to diagnose and treat quickly to preserve spinal stability and neurological function. In a case report in 1946, a 76-year-old patient with septicemia was diagnosed with multifocal discitis caused by *S. warneri* [21]. The patient had previously undergone surgery to drain an abscess in the psoas muscle, from which the infection of the disc space could have arisen. Diagnostic tests, including bone scintigraphy and MRI, showed abnormalities in the affected discs and vertebrae. A needle biopsy confirmed the diagnosis of discitis by detecting the presence of *S. warneri*. This case highlights the importance of vigilant monitoring and early treatment of discitis to prevent complications [21].

### 3.7. Botryomycosis

Botryomycosis (or bacterial pseudo-mycosis or pyoderma vegetans) is a rare chronic infection caused by low-virulence bacteria. It can involve skin and, rarely, internal organs, usually occurring after injury or surgery. *Staphylococcus aureus* appears to be the main etiological agent. However, recently, Yendo et al. (2021) highlighted a particular case of cutaneous botryomycosis caused by *S. warneri* in a healthy 58-year-old woman [53]. The patient had a slow-growing lesion on her right foot. Examination revealed multiple open fistulae with purulent exudative material. A biopsy showed chronic cutaneous granulomatous inflammation with histiocytes, plasma cells and lymphocytes, with the presence of Gram-positive filamentous structures. Further analysis identified *S. warneri* as the etiological agent of the specific botryomycosis. This case is an interesting example of an unusual presentation of botryomycosis and, noticeably, it was caused by *S. warneri* [53].

### 3.8. Neonatal Intensive Care Unit (NICU)

Cimiotti et al. (2007) investigated the relationship between hand hygiene practices and healthcare-associated infections in ill newborns [10]. The study analyzed data from 2935 neonatal admissions and 119 nurses and focused on *S. warneri* isolated from newborns with bloodstream infections and the hands of nurses who cared for these infected infants. Between the 647 bacterial isolates associated with clinical infections in infants, *S. epidermidis* was the most prevalent (151 cases), followed by *S. warneri* (17 cases). These findings suggest a potential role of *S. warneri* in neonatal infections and emphasize the importance of proper hand hygiene practices to prevent transmission [10].

### 3.9. Cerebrospinal Fluid Shunt Infections (CSF)

*S. warneri* has rarely been described in cerebrospinal fluid (CSF) shunt infections. The ventriculoperitoneal (VP) shunt is a cerebral shunt for the treatment of hydrocephalus, removing the excess of cerebrospinal fluid. Martinez-Lage et al. (2010) reported a 17-year-old patient with an intraabdominal pseudocyst due to ventriculoperitoneal shunt infection caused by *S. warneri,* whose ventricular CSF, obtained from the valve reservoir, was sterile, thus causing a significant delay in the diagnosis and treatment of shunt infection. *S. warneri* was detected only after the culture of the removed peritoneal catheter tip [54]. The placement of a ventriculoarterial shunt is considered as a second-line procedure when the standard ventriculoperitoneal shunt and endoscopic third ventriculostomy are not appropriate options [55]. Torre et al. (1992) described the experience of a 12-year-old girl who had hydrocephalus caused by bacterial meningitis at the age of 5 months [1]. The patient presented symptoms of fever and recurrent seizures and, while the analysis of the CSF obtained by lumbar puncture was sterile, the analysis of blood cultures and a cerebrospinal fluid sample obtained from the shunt valve established the presence of *S. warneri* [1]. These cases suggest that *S. warneri* may be clinically significant in CFS shunt infections, highlighting the importance of choosing appropriate pathological specimens for a prompt diagnosis.

### 3.10. Bacterial Ventriculitis

Ventriculitis is the inflammation of the ependymal lining of the cerebral ventricles, usually secondary to infection. Recently, Şimşek et al. (2023) reported the case of a 51-year-old woman with primary bacterial ventriculitis caused by *S. warneri* [56]. The woman did not display typical symptoms, such as fever, neck stiffness, or motor deficits. However, a computed tomography (CT) scan of her brain revealed a hyperdense area in the right lateral ventricle, leading to the diagnosis of ventriculitis. Further examination through lumbar puncture (LP) showed a xanthochromia appearance in the cerebrospinal fluid (CSF), indicating the presence of *S. warneri*. The patient had an infected, ulcerated, and hyperpigmented wound in the left nose–cheek region, which had been present for several years. A histopathological examination indicated the presence of squamous cell carcinoma, ruling out other potential sources of infection. The infected wound and complex lymphatic drainage in the head region were the underlying cause of the ventriculitis [56].

### 3.11. Sepsis

Sepsis is a dangerous and diverse syndrome that occurs when the body overreacts to an infection, resulting in a life-threatening condition. It is a major contributor to infection-related deaths [57]. Ivić I. et al. (2013) reported on a 35-year-old man who developed *S. warneri* sepsis with multiple abscesses [58]. The patient was admitted to the hospital with symptoms including redness, swelling, chills, high fever, and a productive cough with thick sputum. Upon examination, the patient had a subcutaneous abscess on his lower left leg, and blood cultures confirmed the presence of *S. warneri*. The source of the infection was determined to be the subcutaneous abscesses. This case highlights the severity of sepsis caused by *S. warneri* and the importance of prompt diagnosis and treatment to prevent mortality [58].

### 3.12. Urinary Tract Infection

Kanuparthy et al. (2020) presented a case of urinary tract infection (UTI) caused by *S. warneri* in a 65-year-old patient with liver cirrhosis, which could have led to an immunocompromised state [59]. The patient had a history of alcohol abuse and atrial fibrillation. Symptoms included generalized weakness, and painful and frequent urination. Despite no previous urologic problems or UTIs requiring treatment, the urine examination revealed amber-colored urine with leukocytes. Subsequent urine cultures identified CoNS, specifically *S. warneri*, with a concentration of 75,000–100,000 colony-forming units/mL [59].

### 3.13. Meningitis

Meningitis is a condition characterized by inflammation of the membranes covering the brain and spinal cord. It is mostly caused by bacterial infections. Prompt diagnosis of bacterial meningitis is crucial, as it can have serious consequences if left untreated. Incani et al. (2010), reported a 59-year-old patient with symptoms of meningitis caused by *S. warneri*, including fever and productive cough [60]. This occurred in a patient who in principle had hyper-infection syndrome with *Strongyloides stercoralis*, with larvae found in her feces and expectoration. Laboratory analysis of cerebrospinal fluid confirmed the presence of *S. warneri*. The patient was treated with vancomycin and for neurological symptoms of meningitis, and recovered. However, she continued to have fever, cough, and subsequently developed severe gastrointestinal symptoms and pneumonia. In which way *S. warneri* infected the central nervous system is uncertain, but it may have been collected from the skin during external autoinfection by filariform larvae of *S. stercoralis*, and an important factor is that the patient had pruritic lesions on the buttocks during the meningitis process [60].

### 3.14. Veterinary Field

Antibiotics are used in aquaculture, at higher doses than in livestock, thus leaving traces in fish flesh and the aquatic environment. These residues can rapidly spread through feces into water, where they exert selective pressure on surrounding bacteria, sediments and associated microbiota [61].

Espino et al. (2006), reported a rare case of *S. warneri*-associated meningoencephalitis in a dog [30]. Xiao et al. (2022) isolated and identified *S. warneri* from the rare fish *Coreius guichenoti*, which had symptoms such as slowing of movement, hemorrhages in fin rays, ulcers, and skin lesions [34].

Barigye et al. (2007) conducted a study that provided diagnostic evidence of *S. warneri* as a possible cause of bovine abortion [29]. The authors isolated *S. warneri* from placenta, fetal tissue samples, and stomach contents of an aborted bovine fetus, and observed necrotizing lesions in the tongue, lung, and placenta [29].

Musharrafieh et al. (2007) displayed *S. warneri* as a saprophyte in rainbow trout (*Oncorhynchus mykiss*) but, after dysbiosis or interruption of skin homeostasis, this can overgrowth resulting in an inflammatory skin reaction. This condition may lead *S. warneri* to become an indirect pathobiont by enhancing adhesion and biofilm formation of pathogens, such as *V. anguillarum* [31].

These occurrences highlight the capability of *S. warneri* to infect animals as well as humans.

### 3.15. The One Health Approach

One Health is a holistic approach to health that recognizes the existence of close interconnection and interdependence across humans, animals, plants, and shared environments.

Antimicrobial resistance, which increasingly threatens the ability to successfully treat bacterial infection, often hindering or even preventing its resolution, is influenced by the use and abuse of antimicrobials in various contexts: as most classes of antimicrobials used in humans are also used in animals or in plants, it is important to adopt a One Health approach to combat antimicrobial resistance, first of all by preventing the inappropriate use of antibiotics.

The massive administration of antimicrobials—including those of critical importance to humans—to animals in livestock, and the long-term use of these agents to increase food production, are of great emerging importance from the One Health perspective.

The use of antibiotics, such as tetracycline and streptomycin, in agriculture to control bacteria that contaminate fruit is a prime example of what can thwart the One Health approach [62].

### 3.16. Food Field and Correalated Antibiotic Resistance Issues

With the increased use of antimicrobials in the food factory, many cases of food containing antibiotic-resistant CoNS have been reported. In their suggestive article, Silva et al. (2022) reported the presence of multi-drug-resistant and methicillin-resistant CoNS in healthy poultry slaughtered for human consumption [63]. This report suggests that the food chain may represent a transmission route for antibiotic resistance genes.

Staphylococcal food poisoning is one of the most common foodborne illnesses found around the world. This foodborne illness is generally caused by the ingestion of food contaminated with staphylococcal enterotoxins (SEs): *S. warneri*, after producing staphylococcal enterotoxin A (SEA) and enterotoxin D (SED), can induce diseases like acute gastroenteritis [64].

Ready-to-eat foods have also been identified as a source of resistant bacteria; Chajęcka-Wierzchowska et al. (2023) reported on 85 CoNS strains (14 of them *S. warneri*) from various ready-to-eat foods [65]. Food samples were obtained from 11 randomly selected restaurants and bars in Olsztyn, Poland. The study underlined that 78.8% of CoNS isolates were resistant to at least one antibiotic, and 43.5%, especially *S. epidermidis* and *S. warneri*, were multidrug-resistant (resistant to three or more classes of antibiotics). The most commonly observed resistance was to penicillin, followed by erythromycin, cefoxitin, clindamycin, fusidic acid, quinupristin/dalfopristin, gentamicin and rifampicin, while no strain was resistant to ciprofloxacin [65].

In a recent study, a total of 31 CoNS, consisting of *S. warneri* (12), *S. haemolyticus* (6), *S. hominis* (4), *S. saprophyticus* (3), *S. simulans* (3) and *S. xylosus* (3), were isolated from seafood samples. The antibiograms showed high resistance rates against the aminoglycoside antibiotic gentamicin (70.90%), the macrolide antibiotic azithromycin (64.50%), and the tetracycline antibiotic tigecycline (32.20%) [15].

Another alarming example comes from a phenotypic and genotypic study on the antibiotic resistance of *S. warneri* (and *S. pasteuri*) isolated from stuffed mussels. *S. warneri* was detected at a rate of 73.33% (*S. pasteuri* at 26.67%). All *S. warneri* isolates were resistant to amoxicillin/clavulanic acid and erythromycin, while *S. pasteuri* isolates were found to resist amoxicillin/clavulanic acid but not erythromycin. In *S. warneri* isolates, at least three genes responsible for antibiotic resistance (BlaTEM, tetB-6, tetK-8) and up to eight resistance genes (BlaTEM, tetB-6, tetK-8, strA-strB, aphAI-IAB, ermC) were detected. *S. pasteuri* isolates were found to be endowed with blaTEM, strA-strB, and aphAI-IAB resistance genes, but not with tetB-6, tetK-8, and ermC genes. The study demonstrated that antibiotic-resistant *S. warneri* and *S. pasteuri* contaminated stuffed mussels and that *S. warneri* possessed a broader antibiotic resistance profile [16].

In light of these findings, many countries and international agencies have adopted a One Health approach to challenge antimicrobial resistance. The WHO Global Action Plan includes five main objectives, briefly recalled below: (1) improve awareness of the antibiotic resistance phenomenon through education; (2) improve knowledge through surveillance and research; (3) reduce the incidence of infections through their prevention; (4) optimize the use of antimicrobials in human and animal health; (5) increase investment in new therapeutic and diagnostic tools [66].

From all the case reports we have discussed, it can be deduced that *S. warneri* has a tropism in particular for musculoskeletal, implant-associated, and cardiovascular infections, without neglecting other clinical contexts in which the bacterium is establishing itself as a pathogen. Some original data demonstrate the pathological burden of *S. warneri* in orthopedics and highlight that it is beneficial to maintain the aspect of cultural search and identification under observation and continue to monitor epidemiological information over time.

## 4. Pathological Characteristics

*Staphylococcus warneri* exhibits several pathogenic factors with multifactorial mechanism of pathogenicity. The whole genome sequencing of *S. warneri* G1M1F, a multidrug-resistant strain isolated from mouse fecal samples, which induced mastitis, and is deposited at NCBI/GenBank, exhibited the presence of 27 antimicrobial resistance genes (ARGs) and 112 virulence factors in the draft genome [67]. *S. warneri* can produce the aforementioned enterotoxins SEA and SED [6,64]. In a study from the early 1980s, *S. warneri* was found not to possess a rich antimicrobial resistance panel and to be the most susceptible species compared to the other species of CoNS [68]. Then, *S. warneri* was reported to exhibit antibiotic resistance for penicillin and, more in general, β-lactams [12,43,60], and to be multiantibiotic resistant [69], as well as hetero-resistant (i.e., with a phenotype containing subpopulations that show a higher antibiotic resistance compared with the main population) to glycopeptides [70].

The insidiously growing antibiotic resistance characteristics of *S. warneri* culminate in neonatal intensive care units, both in nurses and newborns, where this species is showing an alarming profile of antibiotic resistance [10].

The complete genome sequence of *S. warneri* strain GD01 recovered from a muscle abscess of a pig in South China was profiled [69]. The antibiotic susceptibility test demonstrated that *S. warneri* GD01 was resistant to penicillin, amoxicillin, ampicillin, cefalexin, sulfisoxazole, and even vancomycin. Associations between phenotypes and genotypes were sought in order to identify the molecular basis of resistance and virulence of *S. warneri GD01*. Thus, by analyzing its genome, numerous other genes have been discovered, in addition to the resistance genes, which encode virulence factors, such as adhesins, exoenzymes and iron acquisition proteins [69].

Currently, antibiotic resistance is an emerging issue for *S. warneri,* with considerable clinical significance. Presumably, like the *S. warneri* strain GD01 described in [69], other *S. warneri* isolates may also be equipped with an array of resistance and virulence factors, not to mention that the most invasive strains, by surviving, multiplying, and spreading can gradually enrich their armory of antibiotic resistance factors.

Several studies have reported that *S. warneri* strains can inhibit the growth of other bacteria. Warnericin, a newly discovered bacteriocin contained in the cell-free supernatant from *S. warneri* RB4, was found to inhibit 32 Gram-positive and 6 Gram-negative bacterial strains belonging to various species, as detected through the agar-well diffusion assay [71]. A similar study conducted by other authors on three strains of *S. warneri* (FM10, FM20, and FM30) reported that the bacteriocin warnerin inhibited the growth of many Gram-positive and Gram-negative species [72]. Warnericin RB4, extracted and characterized for the first time by Minamikawa et al. (2005) [71], is an *S. warneri* bacteriocin with a molecular mass of 2958.2 Dalton; it exerts a specific antibacterial activity against thermoacidophil bacteria, such as *Alicyclobacillus* spp. Warnericin RB4 is inactivated by trypsin and actinase E. Nukacin ISK-1, a type A(II) lantibiotic or closely related bacteriocin described in *S. warneri ISK-1*, exhibits an excellent heat stability [73,74,75,76,77]. Nukacin ISK-1 gene is a cluster containing *nukA* (a structural gene), *-M*, *-T*, *-F*, *-E*, *-G* genes, and two open reading frames, ORF1 and ORF7 [78]. While *nukM* and *nukT* are involved in the post-translational modification and secretion of nukacin ISK-1, respectively, *nukF*, -*E* and *G* are responsible for creating a membrane complex for self-protection from nukacin ISK-1. Nukacin ISK-1 is transferred to the extracellular space by NukFEG after being captured by NukH [79]. *S. warneri* ISK-1 carries two plasmids, pPI-1, which encodes for the bacteriocin biosynthesis (i.e.,: nukacin) and immunity genes, and pPI-2, which belongs to the *qacC* plasmid family and contains the disinfectant resistant gene *qacC* [80].

A protein was described (LysWMY) similar to the staphylococcal putative *N*-acetylmuramoyl-l-alanine amidases, constituted by three domains (endopeptidase, amidase and a cell wall recognition), which can lyse cells [81]. Héchard et al. (2005) characterized in *S. warneri* a highly hydrophobic peptide, which inhibits *Legionella* growth [82]. Verdon et al. (2007) demonstrated that warnericin RK and delta-lysin I from *S. warneri* have antimicrobial activity against *Legionella* [83]. In a genome sequencing study on *S. warneri* TRPF4, de Souza Freitas et al. (2020), after bioinformatic analysis, it was revealed that the TRPF4 genome holds two gene clusters responsible for the synthesis of three bacteriocins with antimicrobial activity against a range of microbes, especially with anti-*Legionella pneumophila* [84]. There was also one warnericin RK, two delta-lysins, and a new delta-lysin of 3.48 kDa, identified and presented for the first time by de Souza Freitas et al. in [84].

Bogut et al. (2014), for the first time, reported a *S. warneri* small-colony variant (SCV) from a patient with prosthetic-joint infection (PIJ), *mecA*-positive, with a moderate ability to produce biofilm in vitro, whereas its parent strain was not former [85].

Szczuka et al. (2016) reported that 52% of *S. warneri* isolates had the ability to adhere to 96-well polystyrene microtiter plates and to produce a proteinaceous and polysaccharidic biofilm in vitro [86]: they found that the *S. warneri* isolates presented a high degree of genetic diversity and were not clonally related. Further analysis revealed that the *ica* operon responsible for the synthesis of the polysaccharide intercellular adhesin (PIA) —which is present in nearly all *S. aureus* and in a high number of *S. epidermidis* from orthopedic implant infections [87,88,89,90]—was only present in a minority of biofilm formers.

This suggests that biofilm formation in *S. warneri* is not exclusively dependent on the presence of the *icaADBC* genes, as it is for the two CoNS *S. epidermidis* and *S. lugdunensis* and for the CoPS *S. aureus* [86].

Like *S. aureus* and *S. epidermidis*, *S. warneri* has the ability to produce biofilm on vascular catheters and prosthetic devices, which thus protect bacteria and maintain infections [2,91]. Nevertheless, *S. warneri* is not a strong biofilm producer, like other CoNS, such as *S. epidermidis*, *S. lugdunensis*, and the CoPS *S. aureus* [92,93,94].

In our previous research, we detected only 3 biofilm producers on 26 clinical isolates of *S. warneri* through the Congo red agar (CRA) method (unpublished study). Aureolysin gene of the strain *S. warneri* M (*aurWM*) is involved in the suppression of biofilm formation [88]. Indeed, the *S. warneri* Mau (*aurWM*-deficient mutant) produced a greater amount of biofilm than the wild type [95].

Yokoi et al. (2001) characterized the protease m-PROM, a glutamyl endopeptidase, in *S. warneri* M [96]. *S. warneri* M strain possesses the *proMCD* operon, which is the homologue of the *sspABC* proteinase operon of *S. aureus*. The *proM* and *proC* genes encode an endopeptidase responsible for the proteolytic cleavage of extracellular proteins and for a cysteine protease, respectively [97]. PROM and PROC seem to be unrelated to the biofilm formation [95] and contributed to the processing of Atl_WM_, the major putative autolysin [98], which affected the autolysis in a buffer system [97].

Sun et al. (2021), using comparative genomics analysis, identified three different *sdr* genes (*SdrJ*, *SdrK*, and *SdrL*), located at the same *sdr* locus on the chromosome of different *S. warneri* strains, which encode cell wall-anchored (CWA) proteins belonging to the Clf-Sdr subfamily [99].

Despite the fact that *S. warneri* is not considered as a pathogen but a natural member of the human gut microbiota, it can internalize into intestinal epithelial cells using actin-dependent mechanisms and then make changes to the intestinal physiology [35]. Szczuka et al. (2016) characterized 23 *S. warneri* clinical isolates for the ability to produce biofilm, and to adhere to, invade, and destroy epithelial cells [86]. All the isolates adhered to epithelial cells, 43.5% entered them, and 52% formed a biofilm in vitro. Furthermore, 95% of the *S. warneri* cell-free supernatants displayed a cytotoxic activity with destruction of HeLa cells [86].

The attention towards this new pathogen is evident from the growing number of scientific articles and reports on it, which also result from the improved accuracy of identification techniques, as well as its growing antibiotic resistance.

## 5. Antibiotic Resistance and Antibiotic Therapy

Staphylococci are becoming more and more resistant to antibiotics, thus representing a significant challenge in the management of staphylococcal infections worldwide. The issue of bacterial resistance is particularly critical in the case of multidrug-resistant (MDR) and methicillin-resistant (MR) strains: methicillin resistance is determined by the presence of an additional penicillin-binding protein (PBP2a), encoded by the *mecA* gene; this gene is located on a mobile genetic element called the staphylococcal cassette chromosome (SCCmec), which can be transferred from one strain to another of the same species or even of a different staphylococcal species. Noticeably, SCCmec can also carry genes that confer resistance to other antibiotics [100]. Methicillin-resistant strains are not susceptible to penicillins (like oxacillin) nor other β-lactam antibiotics, including those with β-lactamase inhibitors and carbapenems. Moreover, methicillin resistance often implies resistance to other classes of antibiotics, such as macrolides, lincosamides, aminoglycosides, fluoroquinolones, and sulfonamides. Treating methicillin-resistant infections often requires the use of second-line antibiotics, like vancomycin, daptomycin, or linezolid [101].

Garbacz et al. (2021), in a study conducted in Gdansk (Poland), evaluated the antibiotic resistance of different *Staphylococcus* species isolated from 367 oral microbiological specimens [102]. *Staphylococcus* isolates were identified using matrix-assisted laser desorption ionization–time of flight mass spectrometry (MALDI–TOF MS). Their susceptibility to antimicrobial agents was tested using the disk diffusion method. Fifteen different antimicrobial agents were tested, including oxacillin, cefoxitin, gentamicin, erythromycin, clindamycin, tetracycline, chloramphenicol, ciprofloxacin, trimethoprim/sulfamethoxazole, fusidic acid, linezolid, rifampicin, tigecycline, vancomycin and penicillin G. Susceptibility to vancomycin was determined using the E-test method. The study found that the most detected species was *S. aureus* (coagulase-positive staphylococci), which accounted for 46.4% of the strains. Additionally, 103 CoNS were identified, with *S. warneri* being the most prevalent (45.6%). The study aimed to assess the prevalence of antibiotic resistance in these staphylococcal strains and provide information which was epidemiologically important and significant for both laboratories and clinicians [102]. Overall, the 192 *Staphylococcus* spp. isolates showed varying levels of resistance to different antibiotics. The highest resistance was observed against penicillin (62.5%), followed by gentamicin (51%) and erythromycin (30.7%). Lower levels of resistance were recorded for tetracycline (30.2%), cefoxitin/oxacillin (13.5%), clindamycin (15.1%), trimethoprim/sulfamethoxazole (10.4%), fusidic acid (7.8%), chloramphenicol (4.7%), and ciprofloxacin (2.1%). None of the isolates showed resistance to linezolid, rifampin, tigecycline, and vancomycin. Very interestingly, the study also revealed that CoNS had higher levels of antibiotic resistance compared to *S. aureus* isolates. *S. saprophyticus* exhibited high resistance to penicillin (88.9%), *S. haemolyticus* to erythromycin (84.6%), *S. saprophyticus* to fusidic acid (77.8%), *S. epidermidis* to trimethoprim/sulfamethoxazole (71.4%), *S. saprophyticus* to tetracycline (55.6%), and, notably, *S. warneri* to gentamicin (63.8%). These findings highlight the importance of monitoring anti-biotomeic resistance patterns among *Staphylococcus* spp. to promote effective and timely adequate treatment strategies [102].

Szczuka et al. (2016) reported that the biofilm-forming *S. warneri* strains isolated in Poznan (Poland), carried genes encoding for resistance to various classes of antibiotics, including β-lactams, aminoglycosides, macrolide-lincosamide antibiotics, and streptogramin B (MLS_B_), compared to the biofilm-negative isolates [86]. The study also showed that the *mecA* gene was present in only a few of the biofilm-positive strains. Additionally, genes associated with aminoglycoside resistance and phosphotransferases were found in the biofilm-positive isolates. Biofilm-positive *S. warneri* isolates were shown to have a higher occurrence of antibiotic resistance genes compared to biofilm-negative isolates: the identified genes include *aac*(*6′*)/*aph*(*2′*), *aph*(*3′*)-*IIIa*, *erm*(*A*), *erm*(*C*), *msr*(*A*), and *lnu*(*A*). Furthermore, it was observed that most of the strains tested were capable of forming biofilm but did not possess the *erm*(*B*) genes. The proximity of cells within biofilm structures potentially facilitates the transfer of genes horizontally, consequently leading to the spread of antibiotic resistance. These findings emphasize the presence and transferability of antibiotic resistance genes within *S. warneri* isolates [86].

In the study conducted by Regecová I. et al. (2022), among the 45 isolated strains of *S. warneri* out of the 200 CoNS species from food samples, such as chicken, beef, pork (eastern Slovakia), *Oryctolagus cuniculus* (Košice, Slovakia), *Oncorhynchus mykiss* (Czech Republic), *Scomber scombrus* muscles (from Ireland), and cheese (central Slovakia), resistance to ciprofloxacin and tetracycline was the most common, with 73% of the isolates showing resistance to these antibiotics [6]. In particular, the MIC50 (value expressing the lowest inhibitory concentration of a given antibiotic to which at least 50% of the population is inhibited) of ciprofloxacin was 2.0 mg/L and the MIC90 (value expressing the lowest inhibitory concentration of a given antibiotic to which at least 90 percent of the population is inhibited) was 4.0 mg/L, while for tetracycline the MIC50 was 16.0 mg/L and the MIC90 was 32.0 mg/L. At the same time, intermediate susceptibility to trimethoprim + sulfonamide, and nitrofurantoin was confirmed in all 45 isolates. Strains treated with vancomycin, trimethoprim, trimethoprim + sulfonamide, and nitrofurantoin were found to be the most sensitive overall. Multidrug resistance was identified in 10 isolates and, based on the results on the PCR reaction and subsequent sequencing of the PCR products, the presence of the *mecA* gene was confirmed in four isolates (66.6%) [6]. Antibiotic resistance genes were found to be more common in biofilm-positive than biofilm-negative isolates of *S. warneri* [6].

As previously mentioned, Kanuparthy et al. (2020) reported a urinary tract infection (UTI) case caused by *S. warneri* with high concentration of bacteria. An antibiogram was conducted, revealing resistance to penicillin but sensitivity to several antibiotics, like ciprofloxacin, daptomycin, gentamicin, levofloxacin, linezolide, nitrofurantoin, oxacillin, rifampin and triemethoprim/sulfamethoxadole. Levofloxacin was the antibiotic of choice, and the patient showed clinical benefit from this treatment [59].

The reported cases highlight the importance of considering the patient’s medical history and performing appropriate tests to guide antibiotic therapy and to avoid incorrect antibiotic administration and/or overdose in order to counteract the phenomenon of antibiotic resistance. It is also important to be careful not to use subinhibitory concentrations of antibiotics, as these can favor both the formation of bacterial biofilm and the development of antibiotic resistance [103].

The search for new therapeutic strategies to reduce the use, abuse, and misuse of antibiotics is a new trend in modern medicine, in line with the One Health approach [104].

In this connection, the results of recent studies promise to have a favorable impact on the clinic. Here are some examples. In the study of Bright et al. (2022), nanostructured antibacterial surfaces turned out to be capable of enhancing the effect of commonly used antibiotics, thus allowing reduction in antibiotic concentrations in the prevention or treatment of implant-associated infections [105].

Another innovative approach is to exploit antimicrobial peptides (AMPs), such as melittin, to “revive” antibiotics and restore their effectiveness against resistant bacteria [106].

Before addressing the section on industrial applications, we propose a summary table (Table 3) that collects the reports of diseases caused by *Staphylococcus warneri*.

## 6. Industrial and Commercial Objectives

Several authors have described *S. warneri* as a useful microbe for industrial or commercial purposes.

The excellent lipolytic effects of *S. warneri* and the high levels of lipase secreted in culture supernatants make lipase a major emerging interest. This molecule is used for commercial interests, for example, to reduce the lipid content in fish waste and to impart high-quality characteristics to fermented fishmeal paste [122]. In a previous study, *S. warneri* lipase was immobilized in silica gel and catalyzed the synthesis of the ester in organic solvent to produce flavor esters [123]. Other studies have proposed economical methods for industrial lipase bioproduction and discovered new lipases of biotechnological interest. The importance of the biotechnological perspective of lipase production by *S. warneri* in a bioreactor has been previously described [125,126,127]. Volpato G. et al. (2010) purified and separated three new lipases from the crude extract of *S. warneri* using other lipases covalently linked to an adsorption matrix via specific lipase–lipase interactions [128]. Rech F.R. et al. (2011) showed positive effects of polydimethylsiloxane (PDMS) on oxygen volumetric mass transfer coefficient (k_L_a) in *S. warneri* EX17 cultivation and on lipase production for bioreactor cultures [129,130]. An efficient one-step method was proposed to purify and immobilize *S. warneri* EX17 lipase using hydrophobic supports with the aim of obtaining high amounts of this lipase to use in industrial applications [131].

Besides the importance of lipases, the aflatoxin B_1_, observed in *S. warneri* bacterial culture and cell lysate, and its capability for biodegradation are other attractive properties [132]. Pant N.C. et al. (2018, 2019) observed that the sperm agglutinating factor (SAF) from *S. warneri* has spermicidal activity in vitro and contraceptive effectiveness in vivo; consequentially, the authors suggest applying SAF as a vaginal contraceptive [133,134].

Wang M. et al. (2021) improved the flavor quality of fermented meat rice, a typical Chinese food, during its fermentation, by inoculation with *Lactobacillus plantarum* C7 and *Staphylococcus warneri* S6, thus preventing the growth of pathogenic microorganisms [135]. In a recent study, Hawari F.L. et al. (2023) proposed a mix of epidermal commensal bacteria for skincare purposes. *S. warneri* MBF02-19J strain was used, among the bacteria of that mix, for its well-known qualities in producing the bactericidins nukacin ISK-1 and warnericin RK, two antimicrobial peptides active against several pathogens, especially *Legionella* [136].

Bacteriocins, which represent the weapons of bacteria used to kill competing bacteria, are becoming important for industrial implications as bio-preservatives in the food trade while, in orthopedic implantology and, more generally, in the medical device industry, they are being evaluated to prepare antimicrobial surfaces for anti-infective biomaterials.

## 7. Materials and Methods

The first systematic research was conducted on 18 September 2023, in Web of Science, where references with the term “warneri” in the title were searched, resulting in 127 articles, 2 replies, and 1 research poster. The search was extended to Schoolar Google and PubMed (using the criterion mentioned above plus the terms “antibiotic” OR “resistance”), obtaining eight more articles (n = 138). All articles have been downloaded and reviewed. The screening was conducted by selecting only studies written in English, open access articles or via access with the credentials provided by the Rizzoli Orthopedic Institute and the University of Bologna. Then, another skimming was conducted, excluding articles not related to *S. warneri* (e.g., *Cattleya warneri*) (n = 4) or not suitable for the purpose of the review (n = 34), resulting in 100 articles. Moreover, additional articles were searched in PubMed to investigate topics not merely associated with *S. warneri* (n = 36). Ultimately, 136 items were used to conduct the review.

## Figures and Tables

**Table 1:** Misidentification of biochemical tests amended by automated ribo-printing results.

ID	Biochemical Identification	Ribo-Printer^®^ Identification
*cra*2109	*S. warneri*	*S. aureus*
*cra*2844	*S. warneri*	*S. haemolyticus*
*cra*2520	*S. warneri*	*S. aureus*
*cra*2525	*S. warneri*	*S. aureus*
*cra*2981	*S. warneri*	*S. pasteuri*
*cra*3488	*S. warneri*	*S. aureus*
*cra*3882	*S. warneri*	*S. aureus*
*cra*1302	*S. epidermidis*	*S. warneri*
*cra*1371	*S. epidermidis*	*S. warneri*
*cra*1372	*S. epidermidis*	*S. warneri*
*cra*2845	*S. haemolyticus/S. warneri*	*S. warneri*
*cra*3349	*S. warneri/E. aerogenes*	*S. warneri*
*cra*3487	*S. aureus*	*S. warneri*
*cra*3500	CoNS	*S. warneri*
*cra*3665	*S. simulans*	*S. warneri*

**Table 2:** Origin of 48 *S. warneri* clinical isolates from the Rizzoli Orthopedic Institute collection.

ID	POLY	IRI	IM
*cra*1302		√	IF
*cra*1371		√	K
*cra*1372		√	K
*cra*1408		√	IF
*cra*1463		√	IF
*cra*1464		√	H
*cra*1533		√	K
*cra*1567			-
*cra*1586		√	H
*cra*1621			-
*cra*1642		√	TG
*cra*1665			-
*cra*1760		√	H
*cra*1880			-
*cra*1912		√	H
*cra*2005		√	H
*cra*2017	√	√	K
*cra*2125	√	√	K
*cra*2126	√		-
*cra*2191			-
*cra*2210	√	√	H
*cra*2222		√	H
*cra*2255		√	H
*cra*2285			-
*cra*2370		√	IF
*cra*2405	√		-
*cra*2476-2	√	√	K
*cra*2514		√	IF
*cra*2545			-
*cra*2558		√	H
*cra*2776		√	H
*cra*2796		√	H
*cra*2805		√	H
*cra*2845		√	H
*cra*2921		√	IF
*cra*2959		√	H
*cra*2975		√	IF
*cra*3060		√	H
*cra*3064		-	-
*cra*3068		√	H
*cra*3078		√	H
*cra*3294		√	H
*cra*3349	√	√	H
*cra*3487	√	√	A
*cra*3500	√	√	H
*cra*3519			-
*cra*3665	√		-
cra4141	-	√	K

POLY: polymicrobial; IRI: implant-related infection; IM: type of implant material; H: hip prosthesis; K: knee prosthesis; A: arm prosthesis; IF: internal fixation system; TG: tissue graft.

**Table 3:** Cases reported of diseases caused by *Staphylococcus warneri*.

Pathology	Reference
Bacteremia	[18,20,86,107,108]
Native valve endocarditis	[18,22,23,26,39,45,46,47,48,49,50,51,109,110,111,112,113,114]
Orthopedic infection	[24,43,52,85,107,109,112,115,116]
Discitis	[21]
Botryomycosis	[53]
Neonatal and pediatric field	[10,20,112,117,118]
Cerebrospinal fluid shunt infections (CFS)	[1,2,4,54,55,118]
Bacterial ventriculitis	[56]
Sepsis	[11,57,58]
Urinary tract infection (UTI)	[59]
Meningitis	[60]
Medical devices (catheters and vascular graft)	[18,114,115]
Veterinary field	[30,31,34,67,69,109,110,111,119,120,121]
Food field	[6,37,40,64,122,123,124]

## Data Availability

The data presented in this study are available upon request from the corresponding author S.R.
